# Application of equine herpesvirus-1 vaccine inactivated by both formaldehyde and binary ethylenimine in equine

**DOI:** 10.14202/vetworld.2021.1815-1821

**Published:** 2021-07-15

**Authors:** Fatma F. Warda, Hala El Sawy Ahmed, Nermeen G. Shafik, Christine A. Mikhael, Heba M. G. Abd-ElAziz, Walaa A. Mohammed, Eman A. Shosha

**Affiliations:** 1 Agriculture Research Center, Veterinary Serum and Vaccine Research Institute, Abbasia, Cairo, Egypt; 2 Agriculture Research Center, Central Laboratory for Evaluation of Veterinary Biologics, El-Seka-Baida Street, Abbasia, Cairo, Egypt; 3 Department of Microbiology and Immunology, Faculty of Veterinary Medicine, New Valley University, El-Khargia- New Valley governorate, Egypt

**Keywords:** binary ethylene imine, equine herpesvirus-1, formaldehyde, immunogenicity, inactivated vaccine

## Abstract

**Background and Aim:**

Equine herpesvirus-1 infection in horses causes a wide range of manifestations affecting the respiratory tract. The virus can cause serious economic losses through sporadic abortion in pregnant mares, perinatal death, respiratory disease in young foals. This study was designed to prepare inactivated equine herpesvirus-1 (EHV-1) vaccine using both 0.005 M binary ethylenimine (BEI) and 0.0006% formaldehyde (FA) to decrease the use of BEI and provide a good immunological response. The efficacy, safety, and duration of immunity of the prepared inactivated EHV-1 vaccine were evaluated.

**Materials and Methods:**

The prepared FA/BEI-inactivated EHV-1 vaccine was adjuvanted with Alhydrogel and then evaluated by inoculation into guinea pigs, followed by comparison with the commercial inactivated EHV-1 vaccine. These two vaccines were evaluated by testing the safety and immunogenicity in horses classified into two groups. Group A was vaccinated with two doses of the prepared vaccine at a 4-week interval, while Group B was vaccinated with two doses of the commercial vaccine only. Anti-EHV-1 antibodies were detected in horse serum using enzyme-linked immunosorbent assay (ELISA) and virus neutralizing test (VNT).

**Results:**

Regarding the time required to inactivate EHV-1 vaccine, this was decreased using 0.005 M BEI and 0.0006% FA from 24 to 8 h. ELISA in Group A horses demonstrated a significant increase in EHV-1 antibody titer at 2 weeks after the booster dose compared with that for the pre-booster one, from 485 to 855 antibody titer, which then peaked at 1240 in the 3^rd^ month post-vaccination; after that, it began to decline gradually until the 6^th^ month. Meanwhile, in Group B, the ELISA reading increased from 420 to 790 and then peaked at 1215. The VNT mean in Group A increased from 1.1 to 2.5 within 2 weeks after administration of the booster dose, while in Group B it increased from 0.8 to 2.1. Moreover, ELISA in Group A pigs indicated mean antibody titers at the 3^rd^ week post-inoculation of 576 for Group A and 554 for Group B.

**Conclusion:**

The inactivated EHV-1 vaccine, with fewer chemicals, was prepared in a shorter time. It is safe and also more potent to protect horses for up to 6 months against EHV-1 infection than the commercially produced vaccine.

## Introduction

Equine herpesvirus-1 (EHV-1) infection in horses, commonly arising as sporadic outbreaks globally causes a wide range of manifestations affecting the respiratory tract [1,2]. The virus can cause serious economic losses through sporadic or stormy abortion in pregnant mares, perinatal death, respiratory disease in young foals, and is also on rare occasions associated with neurological disorders [1,3]. EHV-1 has lifetime latency, so it can reactivate causing clinical disease during periods of stress and corticosteroid administration [4,5]. EHV-1 is a member of the family *Herpesviridae*, subfamily *Alphaherpesvirinae*, and genus *Varicellovirus* [1,6]. The virus has nine serotypes, with 1-5 affecting domestic horses, but 6-9 affecting wildlife [1,7].

EHV-1 is prevalent in Egypt. It is diagnosed by intranuclear eosinophilic inclusion bodies in the aborted fetal tissues, enzyme-linked immunosorbent assay (ELISA) used on animals of all infected ages and sexes, as well as polymerase chain reaction on aborted fetal tissue [8]. As EHV-1 is present in different localities within the Egyptian governorates, vaccination is the most effective strategy to prevent and control outbreaks of this disease, especially using the inactivated vaccine in endemic areas, along with good animal management. An inactivated vaccine has many advantages compared with a live attenuated one, in that it is safer and has differentiating infected from vaccinated animals compatibility [9]. Efficient vaccination against EHV-1 should induce both humoral and cell-mediated immune responses [10]. Systemic immunity influences the efficacy of vaccination and its protective effect against EHV-1 [11,12]. The serological condition of mares should involve a low ELISA titer before vaccination, with an inactivated vaccine to give a good immune response [13], as a poor immune response to vaccination can enhance the severity of disease after infection of vaccinated animals [14]. Whole inactivated EHV-1 vaccines are the most common commercially available vaccines, which provide varying levels of protection against EHV-1 [15]. Equine immunization with antigens requires a potent adjuvant to stimulate a protective immune response [10,16]. In classical EHV-1 vaccine, the virus is inactivated by binary ethylenimine (BEI), which causes complete viral inactivation within 24 h with a final concentration of 0.008 M. Moreover, vaccines inactivated with formaldehyde (FA) can protect against EHV-1 [17]. However, the use of FA alone as an inactivator can alter viral immunogenicity through destruction of viral structure [18] and BEI also has the limitation of requiring a long inactivation time (24 h), in addition to its carcinogenicity [19]. A partially purified, FA-inactivated EHV-1 vaccine was subsequently reported to provide a good humoral response in horses and partial protection against clinical signs of disease. In addition, viral inactivation with aziridines became an available option, with BEI being used in particular [20]. Simultaneous virus inactivation using two inactivator substances was carried out, and the synergistic effect of FA with BEI was studied [19]. Adjuvants are modulators used in combination with a specific antigen to reduce its volume and to enhance its immunogenicity. Alhydrogel enhances the immune response by slowing the release of the viral antigen [21].

The study aimed: (1) To decrease the use of BEI to avoid its carcinogenicity, (2) to shorten the time required to achieve complete EHV-1 inactivation and accordingly save time in vaccine production, and (3) to evaluate the efficacy, safety, and duration of immunity of the prepared inactivated EHV-1 vaccine.

## Materials and Methods

### Ethical approval

All animals were housed under hygienic measures and care following the animal ethics. The examination and sampling were done without causing any exertion or hurt to the animals. This study was carried out according to the regulation and procedures approved by the ethics committee on animal experimentation of Veterinary Serum and Vaccine Research Institute (VSVRI), Abbasia, Cairo, and the guide for the care and use of animals (National Institute of Health Publication, NO.8023).

### Study period and location

The study was conducted in August 2019 at Equine Vaccine Research Department, Veterinary Serum and Vaccine Research Institute, Abbasia, Cairo, Egypt.

### Virus, vaccine, and antisera

Freeze-dried locally isolated EHV-1 of Vero cells at second passage (VEP_2_) [22] was used for experimental vaccine preparation and the commercial vaccine. Freeze-dried rabbit anti-EHV-1 and 4 sera were used for virus identification.

### Horses, guinea pigs, and mice

Eight healthy adult horses with low antibody titers against EHV-1 [23] were used to evaluate the potency of the prepared vaccine. Two pregnant mares at the last third of pregnancy were used to test the safety. In the case of guinea pigs, three groups (five animals/groups) were used to evaluate the potency of the prepared inactivated EHV-1 vaccine. Regarding mice, 15 pregnant mice were used in the safety test of the prepared vaccines, while 45 albino Swiss mice were used in the challenge test[24].

### African green monkey kidney cell line (Vero)

Vero cells were maintained and grown in Eagle’s minimal essential medium supplemented with 10% newborn calf serum, 100 IU/mL penicillin sodium, and 100 mg/mL streptomycin, and then used for EHV-1 propagation, virus suspension, titration, and vaccine preparation [25]. Titration of EHV-1 was carried out using the microtiter plate technique and the titer was expressed as log_10_ TCID_50_/mL [26].

### Preparation of viral suspension

EHV-1 seed virus of VEP_2_ was propagated on Vero cells for three successive passages (VEP_5_). The vaccine virus fluid was titrated and the titer was calculated as TCID_50_/mL [27]. The prepared viral suspension with a titer of 7.5 log_10_ TCID_50_/mL was inactivated by both BEI [20] at a final concentration of 0.005 M and FA (37-41%) [27] at a final concentration of 0.0006% at 37°C for 8 h with continuous stirring [19]. Sodium thiosulfate and sodium bisulfate with a final concentration of 2% [7,20] were added to stop the action of BEI and FA. In addition, another part of the viral suspension was inactivated by BEI only at a final concentration of 0.008 M at 37°C for 24 h with continuous stirring [7]. Inactivated EHV-1 suspensions were mixed with 20% aluminum hydroxide gel (Alhydrogel, Superfos Biosectoe, Denmark), as an adjuvant and stirred on a magnetic stirrer for homogenization for 24 h at 4°C to ensure virus adsorption. The pH was adjusted to 7.5, after which thiomersal was added as a vaccine preservative at a final concentration of 0.001% and then distributed into sterile vials (2 mL/vial) [19].

### Vaccine quality control

#### Detection of residual virus activity and sterility test

Detection of residual virus activity and sterility test was performed on the inactivated virus fluid just after the inactivation process to ensure complete virus inactivation [7,27]. Undiluted inactivated EHV-1 was inoculated on the chorioallantoic membrane (CAM) of specific pathogen-free embryonated chicken eggs (ECE) for 11-13 days and then incubated at 37°C for 5 days with daily examination. The absence of pock lesions on CAM was confirmed [6,25,28]. The sterility test was applied to inactivated virus suspensions as well as the final products. The samples were then cultured on different media to rule out bacterial, fungal, and mycoplasma contamination [24].

### Safety test in mice and horses

The safety test was performed on the final vaccine product. One group of pregnant mice was inoculated subcutaneously (S/C) with the prepared vaccines at 0.2 mL/mouse with a contact control group [24,29]. In addition, two pregnant mares at the last third of pregnancy were used to test the safety of the prepared vaccine [25]. The groups of mice and mares were kept under observation under good hygienic conditions for 2 weeks.

### Potency test in guinea pigs, mice, and horses

Fifteen seronegative guinea pigs were divided into three groups (five animals/groups). Group A was inoculated S/C with 2 mL of the prepared inactivated EHV-1 vaccine. Group B was inoculated with the prepared vaccine (0.008 M BEI only). Group C was kept without any inoculation as a control group. After 3 weeks, Groups A and B were inoculated with a booster dose. Five weeks post-inoculation, serum samples were collected from all groups and then tested for EHV-1 antibodies by indirect ELISA [25]. In mice, 30 seronegative mice were divided into three groups (ten mice/group) and inoculated S/C with 0.2 mL of inactivated EHV-1 vaccine with Alhydrogel adjuvant (each of the two EHV-1 vaccines was inoculated into a mouse group) [19,29]. Group A was inoculated with the prepared vaccine (0.005 M BEI/0.0006% formalin). Group B was inoculated with the commercial vaccine (0.008 M BEI only). Group C was kept as a control under the same conditions. After 1 week, Groups A and B were inoculated with a booster dose, and then all mouse groups were challenged intranasally (I/N) 10 days post-inoculation by 45 m 5 living EHV-1 at 7.5 log_10_TCID_50_/mL. Two weeks later, two mice from each group were sacrificed at intervals of 3, 5, 7, and 9 days post-challenge. Then, 10% of liver and lung suspensions from the sacrificed mice were inoculated on CAM of ECE to detect the role of the vaccine in reducing virus circulation and excretion [19]. All groups were kept under observation in hygienic conditions for 5 weeks in the case of guinea pigs and 2 weeks in mice.

In the case of horses, eight local breeds, 3-5 years old with low antibody titers (≤4 neutralizing antibodies) against EHV-1, were divided into three groups. In Group A, three horses were inoculated with 2 mL of the prepared inactivated EHV-1 vaccine (0.005 M BEI/0.0006% formalin) [25]. In Group B, three horses were inoculated with the recommended dose of commercial vaccine (0.008 M BEI only) [26]. In Group C, two horses were kept as a control under the same experimental conditions. Each group was inoculated with two doses of the tested commercial and prepared EHV-1 vaccines applied 1 month apart. Each dose was injected I/M in the lower third of the neck [25]. Serum samples were collected from each group at different intervals for monitoring the immune response using ELISA and virus neutralizing test [6,25].

### Indirect ELISA and neutralization test

Single dilution indirect ELISA was performed on horse and guinea pig sera and the ELISA readings were converted to antibody titer in serum, in accordance with a previous report [30].

Regarding the neutralization test, serum samples from the vaccinated groups were collected weekly and inactivated in a water bath at 56°C for 30 min, followed by testing. The antibody titer was expressed as the neutralization index (NI), as reported previously [31,32]. Furthermore, identification of the selected vaccine seed virus EHIV-l VEP_2_ was carried out by a virus neutralization test using reference antiserum against EHV-1 and -4 [14].

### Statistical analysis

The resulting data were analyzed by one-way analysis of variance in horses. The difference between the mean values was assessed using the least significant difference as p≤0.05 and t-test in guinea pigs as p≤0.05, using SAS/STAT Guide for Personal Computers, version 9 [33].

## Results

### Identity test

Vaccine seed virus (VEp_5_) was completely neutralized (100%) by reference antisera against EHV-1.

### Virus inactivation

The infectivity titer of EHV-1 VEp_5_ was 7.5 log_10_TCID_50_/mL. This titer is recommended for the preparation of inactivated vaccines [34]. Then, the virus was inactivated with both BEI at a final concentration of 0.005 M and FA at a final concentration of 0.0006% at 37°C for 8 h with continuous stirring [19]. Sodium thiosulfate and sodium bisulfate at a final concentration of 2% were added to stop the action of BEI and FA.

### Sterility test

The prepared EHV-1 vaccine was proven to be free from any contamination by bacteria, fungi, or other viruses.

### Safety test

Inoculation of the prepared EHV-1 vaccine in susceptible pregnant mares and mice did not show any notable clinical signs or unfavorable reactions or deaths. This supported that the tested vaccine was safe to be used. In addition, all inoculated horses showed a normal body temperature and no undesirable local or systemic reactions were observed following primary and booster injections.

### Efficacy of vaccine

**Figure-1:**
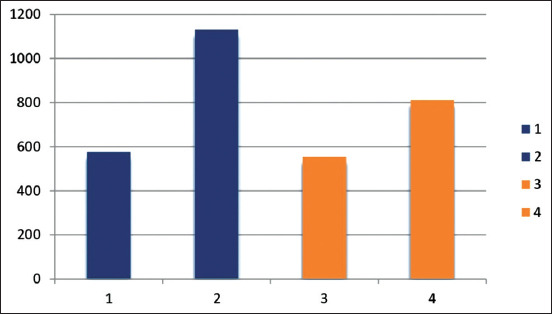
Equine herpesvirus-1 (EHV-1) enzyme-linked immunosorbent assay antibody mean titers in guinea pigs inoculated with inactivated EHV-1 vaccines. 1: Mean titer of Group (A) after 3WPI. 2: Mean titer of Group (A) after 5WPI. 3: Mean titer of Group (B) after 3WPI. 4: Mean titer of Group (B) 5WPI.

The potency of the prepared vaccine in guinea pigs compared with that of the commercial vaccine was evaluated by ELISA (Table-1 and Figure-1). Group A guinea pigs inoculated with 2 mL of prepared inactivated EHV-1 vaccine exhibited antibody titers detectable at the 3^rd^ week post-inoculation, with mean ELISA titers of 576 for Group A and 554 for Group B. A significant increase in the antibody titer (two-fold) was observed 2 weeks after the second dose, with mean ELISA titers of 1150 for Group A and 812 for Group B.

**Table-1:** EHV–1 ELISA antibody titers in guinea pigs inoculated with inactivated EHV-1 vaccines.

Guinea pig/WPI	EHV-I Antibody titer						

	Group (A)			Group (B)			Group (C)
		
	Time of booster dose			Time of booster dose			Average along test
	
	0	3	5	0	3	5	
1	0	620	1300	0	500	660	0
2	0	560	1200	0	610	950	0
3	0	600	1200	0	590	850	0
4	0	520	950	0	600	900	0
5	0	580	1100	0	520	700	0
Mean	0	576	1150	0	554	812	0

Group (A): Inoculated with the prepared inactivated EHV-1 vaccine. Group (B): Inoculated with the commercially produce inactivated EHV-1 vaccine. Group (C): Kept without inoculation as control at the same conditions of the experiments. ELISA readings was converted to antibody titer in serum. EHV-1=Equine herpesvirus-1, ELISA=Enzymelinked immunosorbent assay

### Immunogenicity testing

**Figure-2:**
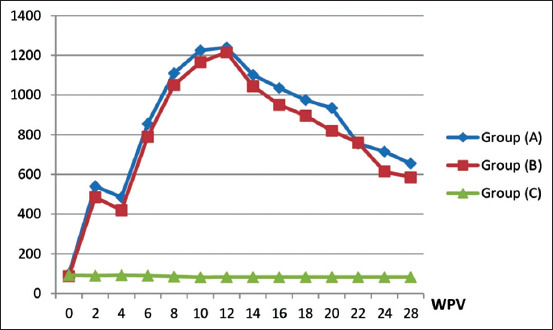
Equine herpesvirus-1 (EHV-1) enzyme-linked immunosorbent assay antibody mean titers n horses vaccinated with the inactivated EHV-1 vaccines. Group (A): Inoculated with the prepared inactivated EHV-1 vaccine. Group (B): Inoculated with the commercially produce inactivated EHV-1 vaccine. Group (C): Kept without inoculation as control at the same conditions of the experiments. WPV=Week post-vaccination.

**Figure-3:**
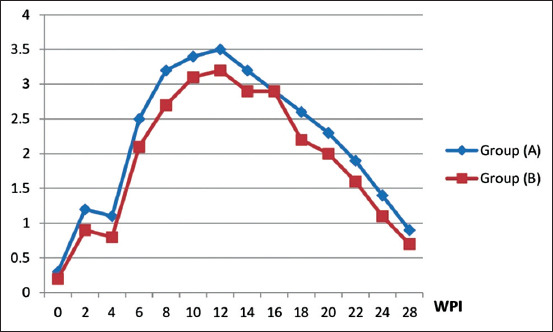
Equine herpesvirus-1 serum neutralizing index in vaccinated horses. Group (A): Inoculated with the prepared inactivated EHV-1 vaccine. Group (B): Inoculated with the commercially produce inactivated EHV-1 vaccine. WPI=Week post-inoculation.

The immunization of horses with the prepared and commercial vaccines was analyzed by ELISA (Tables-2 and 3, and Figures-2 and 3). Group A, which was inoculated with 2 mL of inactivated EHV-1 vaccine, exhibited specific detectable EHV-1 antibody titers at 2 week post-vaccination (WPV), with mean ELISA titer of 540 and mean NI of 1.2 in Group A, and corresponding values of 485 and 0.9 in Group B. Antibody titers decreased at the fourth WPV, showing an ELISA titer of 485 and NI of 1.1 in Group A, and corresponding values of 420 and 0.8 in Group B. Following the booster dose at the fourth WPV, there was a significant two-fold increase of antibody titer obtained at the sixth WPV (the 2^nd^ week post-booster), with mean ELISA titer of 855 and NI of 2.5 for Group A, and corresponding values of 790 and 2.1 for Group B, as NI antibody titer increased from 1.5 to 2-fold, the vaccine considered protective after two weeks post-booster **[7]**. High levels of antibodies were gradually increased and reached their peak at the 3^rd^ month post-vaccination (MPV), with mean ELISA titer of 1240 and NI of 3.5 for Group A, and corresponding values of 1215 and 3.2 for Group B. At the fourth MPV, the mean ELISA titer decreased to 1035 and mean NI to 2.9 in Group A, while the corresponding values were 950 and 2.9 in Group B. Moreover, at the sixth MPV, there were great decreases in these values, with mean ELISA titer decreasing to 715 and NI to 1.4 in Group A, while the corresponding values were 615 and 1.1 in Group B.

**Table-2:** EHV-1 ELISA antibody mean titers in horses vaccinated with the inactivated EHV-1 vaccines.

Time of serum testing/ WPV	Group (A)	Group (B)	Group (C)
Pre vaccination	97.5	87.5	92.5
2	540	485	90
4 (B)	485	420	92.5
6	855	790	90
8	1110	1050	85
10	1225	1165	81.5
12	1240*	1215*	82.5
14	1102	1045	82.5
16	1035	950	82.5
18	975	895	82.5
20	935	820	82.5
22	755	760	82.5
24	715	615	82.5
28	655	585	82.5

Group (A): Inoculated with the prepared inactivated EHV-1 vaccine. Group (B): Inoculated with the commercially produce inactivated EHV-1 vaccine. Group (C): Kept without inoculation as control at the same conditions of the experiments. WPV=Week postvaccination. B=Time of booster dose.

* =Peak of ELISA antibody titers. EHV-1=Equine herpesvirus-1

**Table-3:** EHV-1 virus neutralizing index in vaccinated horses.

Time of serum testing/WPV	Group (A)	Group (B)	Group (C)
Pre vaccination	0.3	0.2	0.3
2	1.2	0.9	0.2
4 (B)*	1.1	0.8	0.15
6	2.5	2.1	-
8	3.2	2.7	-
10	3.4	3.1	-
12	3.5*	3.2*	-
14	3.2	2.9	-
16	2.9	2.9	-
18	2.6	2.2	-
20	2.3	2.0	-
22	1.9	1.6	-
24	1.4	1.1	-
28	0.9	0.7	-

Group (A): Inoculated with the prepared inactivated EHV-1 vaccine. Group (B): Inoculated with the commercially produce inactivated EHV-1 vaccine. Group (C): kept without inoculation as control at the same conditions of the experiments. WPV=Week post-vaccination. B=Time of booster dose.

* =Peak of neutralizing index. EHV-1=Equine herpesvirus-1

### Challenge test

Concerning the duration of viral re-isolation from challenged mice as indicated in Table-4, this was significantly shorter in the inoculated mice than in the control group. In Group A, 0% virus isolation [disappearance of the virus] was observed at the 7^th^ day post-challenge (DPC) (Table-4). Meanwhile, in Group B, the virus isolation disappeared at the ninth DPC. In contrast to the control group, 75% and 25% of the re-isolated viruses (Table-4) were identified at the seventh and ninth DPC, respectively. [i.e. liver of mice inoculated in ECE-CAM to detect the presence of pock lesion]

**Table-4:** Equine herpesvirus-1 re-isolation from inoculated mice after challenge test.

Day post-challenge	% of virus re-isolation		

	Group (A)	Group (B)	Group (C)
3	100	100	100
5	75	75	100
7	0	25	75
9	0	0	25

### Statistical analysis

EHV-1 ELISA antibody titers in horses vaccinated with the inactivated EHV-1 vaccines showed a significant result at 0.05 and the p-value =0.00. EHV-1 neutralizing index in vaccinated horses gave a significant value at 0.05 and the p-value =0.015. In addition, in guinea pigs, ELISA antibody titers on inoculation with inactivated EHV-1 vaccines showed a significant result at 0.05 as p≤0.05 in 3 and 5 weeks towards Group A.

## Discussion

The equine industry is economically important in many countries, especially in Egypt and elsewhere in the Middle East. EHV-1 causes respiratory symptoms, abortion in mares, the birth of weak, nonviable foals, and even sporadic paralytic neurologic disease [1-3]. Efficient vaccination in combination with good animal management is the best strategy to prevent and control outbreaks of the disease, especially using the inactivated vaccine in endemic areas [7]. EHV-1 vaccination is important to ensure protective immunity in foals during the 1^st^ weeks after delivery [35]. An effective vaccine requires a preferable antigen and a strong adjuvant to maximize the vaccine potency and enhance the antigen immunogenicity [36].

Our study was designed for the preparation and evaluation of a vaccine for EHV-1 inactivated with a lower amount of BEI to decrease its carcinogenic effect and the use of FA. Specifically, it included 0.005 M BEI and 0.0006% FA, instead of 0.008 M BEI as in the commercial vaccine, to improve the immune response.

The study results show that the prepared vaccine reduced the time needed for vaccine production by decreasing the duration of the inactivation process from 24 to 8 h. The prepared vaccine was safe in pregnant mares and mice that did not show abortion or any abnormal symptoms after vaccination, in agreement with a previous report [6].

The prepared vaccine was potent in vaccinated guinea pigs and mares in comparison with the commercial vaccine, as shown in Tables-1-3 and Figures-1-3, which revealed that the prepared vaccine confers strong protection until the sixth MPV. These results agree with previous reports [7,25,31,37] that demonstrated NI antibody titer in horses increased from 1.5 to 2-fold after two weeks post-booster, so the vaccine is potent and protective. This result also agrees with another study [31] that concluded that horse revaccination against EHV-1 must be carried out every 6 months to maintain protective immunity levels. Challenge test in mice showed EHV-1 re-isolation (Table-4) and confirmed the superior effect of the prepared vaccine as 0% virus isolation (disappearance of the virus) was observed at the 7^th^ DPC, while the commercial vaccine recorded 0% at the ninth DPC. This finding agrees with a previous report [19] in which it was stated that EHV-1 was recovered from the lung of infected mice within 9 days. It also agrees with another report [38] in which it was reported that FA can inactivate EHV-1 for a vaccine that works well and protects against infection and challenge.

## Conclusion

The prepared EHV-1 vaccine, inactivated by both BEI at 0.005 M and FA at 0.0006%, was rapidly inactivated within 8 h without affecting viral immunogenicity. Furthermore, it was safe and effective through providing protective immunity in vaccinated guinea pigs and horses for up to 6 months. In addition, statistical analysis showed significant results in horses and guinea pigs vaccinated with the prepared inactivated EHV-1 vaccine compared with the commercial tested vaccine. This work thus involved a successful, effective trial to produce inactivated EHV-1 vaccine with a lower amount of BEI than that present in the commercial vaccine, to avoid its carcinogenic effect. This study also used FA to accelerate the virus inactivation step for more rapid vaccine production and to ensure its efficacy in horses.

## Authors’ Contributions

FFW, HEA, and EAS: Designed this study and performed the experimental tests. NGS, HMGA, and WAM: Collected samples. EAS and CAM: Revised the manuscript. All authors read and approved the final manuscript.

## References

[1] MSD (2021). Equine Herpesvirus Infection.

[2] Pusterla N, Mapes S, Akana N, Barnett C, Mackenzie C, Gaughan E, Craig B, Chappell D, Vaala W (2016). Prevalence factors associated with equine herpesvirus Type 1 infection in equids with upper respiratory tract infection and/or acute onset of neurological signs from 2008 to 2014. Vet. Rec.

[3] Mesquita A.L.P, Andressa F.A, Dennis A, Zanattoa S.I, Eleice M.S, Maria D, Eliana M, Claudia M, Paulo C, Enio M (2017). Equine herpesvirus Type 1 induces both neurological and respiratory disease in Syrian Hamster. Vet. Microbiol.

[4] Foote C.E, Love D.N, Gilkerson J.R, Wellington J.E, Whalley J.M (2006). EHV-1 and EHV-4 infection in vaccinated mares and their foals. Vet. Immunol. Immunopathol.

[5] Barrandeguy M, Vissani A, Olguin C, Becerra L, Mino S, Pereda A, Oriol J, Thiry E (2008). Experimental reactivation of equine herpesvirus-3 following corticosteroid treatment. Equine Vet. J.

[6] OIE (Office of International des Epizooties) (2019). Manual of Standards Diagnostic Tests and Vaccines of Terrestrial Animals, Equine Rhinopneumonites. Ch. 12-18.

[7] Nehal S.S (2006). Preliminary Trials for Production of Equine Viral Abortion Inactivated Vaccine.

[8] Salib A.F, Magda A.K, Hassan H.M, Said S.F (2016). Using indirect ELISA and PCR for the diagnosis of equine herpes virus-1 (EHV-1) infection in Egypt. J. Vet. Med. Res.

[9] Van Riijn P.A, Maris-Veldhuis M.A, Grobler M, Wright I.M, Erasmus B.J, Maartens L.H, Potgieter C.A (2020). Safety and efficacy of inactivated African horse Sickness (AHS) vaccine formulated with different adjuvants. Vaccine.

[10] Paillot R, Case R, Ross J, Newton R, Nugent J (2008). equine herpes virus-1:Virus, immunity and vaccines. Open Vet. Sci. J.

[11] Wagner B, Perkins G, Babasyon S, Freer H, Keggan A, Goodman L.B, Glaser A, Torsteinsdottir S, Svansson V, Bjorndottir S (2017). Neonatal immunization with a single IL-4/antigen dose induces increased antibody responses after challenge infection with equine herpesvirus Type-1 (EHV-1) AT weanling age. PLoS One.

[12] Schabel C.L, Babasyan S, Rollins A, Freer H, Wimer C.L, Perkins G.A, Raza F, Osterrieder N, Wagner B (2019). An equine herpesvirus type 1 (EHV-1) Ab4 open reading frame (ORF) 2 deletion mutant provides immunity and protection from EHV-1 infection and disease. J Virol.

[13] Attili A, Colognato R, Preziuso S, Moriconi M, Valentini S, Petrini S, De Mia G, Cuteri V (2020). Evaluation of three different vaccination against EHV1/EHV4 infection in Mares:Double blind, randomized clinical trial. Vaccine.

[14] Huisman W, Martina B.E, Rimmelzwaan G.F, Gruters R.A, Osterhaus A.D (2009). Vaccineinduced enhancement of viral infections. Vaccine.

[15] Kapoor S, Sharma H, Singh M, Kumar P, Ranjan K, Kumari A, Khirbat R (2014). Equine herpesviruses:A brief review. Adv. Animal Vet. Sci.

[16] Safaa A.W, Hussin M.G (2012). Immune response of horses vaccinated with tissue culture inactivated EHV-1 oil (Montanide ISA-70) adjuvanted vaccine. Egypt. J. Agric. Res.

[17] Skinner G.R, Davies J (2000). Efficacy of an inactivated vaccine for equine herpesvirus Type 1 in a novel hamster model. Intervirology.

[18] Tano Y, Shimizu H, Martin J, Nishimura Y, Simizu B, Miyamura T (2007). Antigenic characterization of a formalin-inactivated poliovirus vaccine derived from live attenuated Sabin strains. Vaccine.

[19] Fatma F.W, Maha R.A.E, Ibrahim M.M (2016). Enhancing the BEI-inactivation rate of equine herpes virus-1 by formaldehyde. 8^th^ Scientific Conferences of the Egypt Society for Animal Management at 23-27, August.

[20] Bahneman H.G (1990). Inactivation of viral antigens for vaccine preparation with particular reference to the application of binary ethylenimine. Vaccine.

[21] Coffman R.L, Sher A, Seder R.A (2010). Vaccine adjuvants:Putting innate immunity to work. Immunity.

[22] Magda A.K, Maysa H, Safaa A.W, Nehal S.S, Nashwa K.M, Heba E.G, Sohair E, Eman M.E (2013). Prospective studies of equine herpesvirus-1 myeloencephalopathy in Egypt 2012. Ippologia Anno.

[23] Magda A.K, Eman M.E, Nashwa K.M, Safaa A.W, Nehal S.S, El-Kabbany M.M.A, Soliman I.M.A, Magdi D.S, Deidra S.L, Emad M.E, Jeffrey A.T (2011). Characterization of equine influenza virus H3N8 isolated in Egypt in 2008. Ippologia.

[24] OIE (Office of International des Epizooties) (2012). Manual of Standards Diagnostic Tests and Vaccines of Terrestrial Animals, Equine Rhinopneumonites.

[25] Nashwa K.M, Eman M.E, Nehal S.S, Fatma F.Y, El-Kabbany M.M.A, Soliuman I.M.A (2016). Duration of immunity induced by combined vaccine against equine influenza and equine herpes-1. Vet. Med. J.

[26] Reed L.J, Munech H (1938). A simple method of estimating fifty percent endpoints. Am. J. Epidemiol.

[27] Farid A, Reda I, Moussa A.M, Daoud A (1979). The effect of different chemical inactivators on the antigenicity and infectivity of FMD virus. J. Egypt. Vet. Med. Assoc.

[28] Tyerell D.A.J, Valentine R.C (1957). Assay of influenza virus particles by haemagglutination and electron microscopy. J. Gen. Microbiol.

[29] Slater I.D, Gibson J.S, Field H.J (1993). Pathogenicity of thymidine kinase-deficient mutant of EHV-1 in mice and specific pathogen-free foals. J. Gen. Virol.

[30] SuGiura T, Kondo T, Matsumura T, Imagawa H, Kamada M, Ihara T (1997). Evaluation of enzyme-linked immunosorbent assay for titration of antibodies to EHV-1. J. Equine Sci.

[31] Dalia N (2017). Study the Effect of Different Adjuvants on Inactivated Equine Herpes Virus-1 Vaccine.

[32] Hamdy A.E (2016). Preparation of Inactivated EHV-1 with Different Adjuvants, Ph.D. Thesis.

[33] Gomez K.A, Gomez A.A (1984). Statistical Procedures for Agriculture Research.

[34] Mumford J.A, Bates J (1984). Trials of an inactivated equine herpesvirus-1 vaccine:Challenge with a subtype 2 virus. Vet. Rec.

[35] Di Francesco C.E, Smoglica C, De Amicis I, Cafini F, Carluccio A, Contri A (2020). Evaluation of colostral immunity against equine herpesvirus type 1 (EHV-1) in Martina franca's foals. Front. Vet. Sci.

[36] Harold F, Stills J (2005). Adjuvants and antibody production:Dispelling the myths associated with Freund's complete and other adjuvants. ILAR J.

[37] Guo P, Goebel S, Davis S, Perkus M.E, Languet B, Desmet'rae P, Allen G.P, Paoletrt E (1989). Expression in recombinant vaccinia virus of the equine herpesvirus 1 gene encoding glycoprotein gpl3 and protection of immunized animals. J. Virol.

[38] Delrue I, Verzele D, Madder A, Nauwynck H (2012). Inactivated virus vaccines from chemistry to prophylaxis:Merits, risks and challenges. Expert Rev. Vaccines.

